# Posterior insular cortex – a site of vestibular–somatosensory interaction?

**DOI:** 10.1002/brb3.155

**Published:** 2013-07-23

**Authors:** Bernhard Baier, Peter zu Eulenburg, Christoph Best, Christian Geber, Wibke Müller-Forell, Frank Birklein, Marianne Dieterich

**Affiliations:** 1 Department of Neurology, University Medical Centre of the Johannes Gutenberg UniversityMainz, Germany; 2 Department of Neuroradiology, University Medical Centre of the Johannes Gutenberg UniversityMainz, Germany; 3 Department of Neurology and German Vertigo/Dizziness Center IFB, Ludwig-Maximilians-UniversityMunich, Germany

**Keywords:** Insula, lesion, somatosensory system, stroke, vestibular

## Abstract

**Background** In previous imaging studies the insular cortex (IC) has been identified as an essential part of the processing of a wide spectrum of perception and sensorimotor integration. Yet, there are no systematic lesion studies in a sufficient number of patients examining whether processing of vestibular and the interaction of somatosensory and vestibular signals take place in the IC. **Methods** We investigated acute stroke patients with lesions affecting the IC in order to fill this gap. In detail, we explored signs of a vestibular tone imbalance such as the deviation of the subjective visual vertical (SVV). We applied voxel-lesion behaviour mapping analysis in 27 patients with acute unilateral stroke. **Results** Our data demonstrate that patients with lesions of the posterior IC have an abnormal tilt of SVV. Furthermore, re-analysing data of 20 patients from a previous study, we found a positive correlation between thermal perception contralateral to the stroke and the severity of the SVV tilt. **Conclusions** We conclude that the IC is a sensory brain region where different modalities might interact.

## Introduction

Lesion mapping and functional imaging studies suggested that the posterior insula and adjacent operculum (OP) is a main region of the human vestibular cortical network (Suzuki et al. 2001; Emri et al. 2003; Baier et al. 2012; zu Eulenburg et al. 2012; Lopez et al. 2012). In addition, the insular cortex (IC) seems essential for pain encoding, temperature perception, and thermoregulation (Craig 2000; Schreckenberger et al. 2005; Kong et al. 2006). Previous studies suggest an interaction between pain and vestibular stimulation, which has been investigated in patients with central poststroke pain. These patients were treated with vestibular caloric stimulation and showed a significant pain relief (McGeoch et al. 2008, 2009). Recently, further evidence between the vestibular and somatosensory systems was reported (Ferrè et al. 2012). Cold caloric stimulation modulates somatosensory cortical processing. One anatomical interface of this interaction could be the IC (Garcia-Larrea et al. 2010). The approach of the present study focused on a strong hypothesis regarding the predominant role of the IC/peri-insular region in vestibular otolith processing. Therefore, parameters of otolith dysfunction like head tilt (HT), skew deviation and ocular torsion (OT), and the resulting tilt of subjective visual vertical (SVV) as a perceptual parameter were investigated in patients with acute unilateral stroke. The stroke must affect parts of the IC and the peri-insular brain regions. Furthermore, employing data from a previous study we tested whether vestibular and somatosensory abnormalities might interact after IC stroke.

## Material and Methods

### Neurological examination

**Figure 1:**
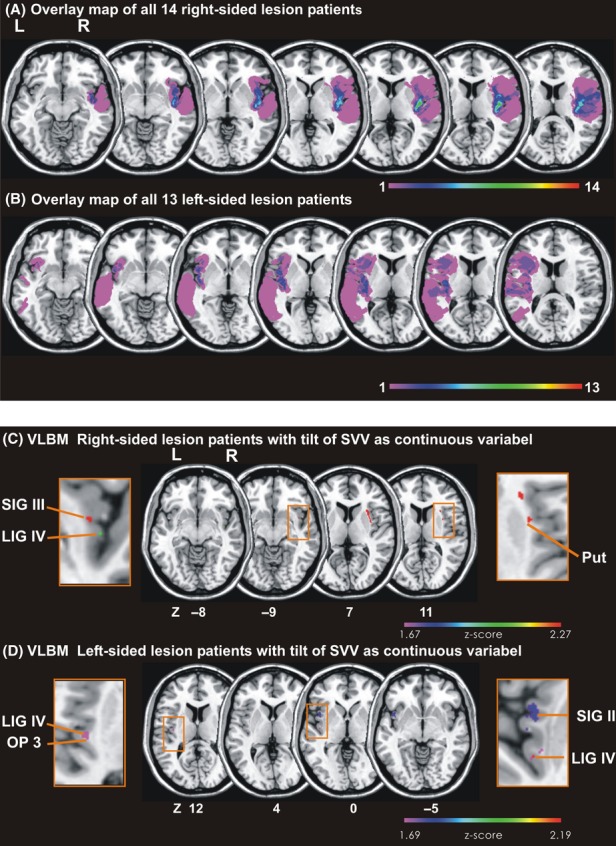
(A) Overlay lesion plots of patients with right-sided lesions. (B) Overlay lesion plot of the patients with left-sided lesions. The number of overlapping lesions is illustrated by different colors coding increasing frequencies from violet (*n* = 1) to red (maximum number) (C). Statistical VLBM analysis of the right-sided lesion patients using tilt of SVV as continuous variable. The key areas covered parts of the short insular gyrus (SIG), the putamen (Put), and small parts of the long insular gyrus (LIG). (D) Statistical VLBM analysis of the left-sided lesion patients using tilt of SVV as continuous variable. The key areas covered partly the SIG, the LIG, and the operculum (OP). L, left side; R, right side; VLBM, voxelwise lesion behavior mapping; SVV, subjective visual vertical.

We investigated a series of 27 patients admitted with an unilateral ischemic stroke affecting the IC (Table 1). As vascular lesions affecting exclusively the IC are extremely rare, we sampled patients with circumscribed damage predominantly to the insular and the opercular peri-IC, which was documented by magnetic resonance imaging (MRI) (13 female, 14 male; mean age 65 years; standard deviation [SD] 12 years) (Fig. 1A and B). Mean time between stroke and testing was 7 days (SD 4 days). Patients with diffuse brain damage or severe communication deficits due to aphasia were excluded. Neglect was assessed in 16 patients by the standardized test battery of the German version of the Behavioural Inattention Test (Fels and Geissner 1987) and in five patients by the bells test (Gauthier et al. 1989). In two of the right-sided and four of the left-sided patients neglect was not tested. Only one patient with right-sided infarction showed neglect. In addition to the actual study, we reanalyzed previous already existing data from 20 patients to show whether there might be an interaction between somatosensory and vestibular parameters (B. Baier, P. zu Eulenburg, C. Geber, R. Rohde, R. Rolke, C. Maihöfner, F. Birklein, M. Dieterich, unpubl. ms; Dieterich and Brandt 1993; Brandt et al. 1994; Rolke et al. 2006). The study was carried out in accordance with the ethical standards laid out in the 1962 Declaration of Helsinki.

**Table 1:** Demographic and clinical data

	Right-brain damage		Left-brain damage	
		
	Normal tilt	Abnormal tilt	Normal tilt	Abnormal tilt
Number	9	5	9	4
Age (years), mean (SD)	63 (10)	68 (14)	64 (16)	69 (9)
Gender (F/M)	5 F, 4 M	4 F, 1 M	4 F, 5 M	0 F, 4 M
Lesion volume (in ccm), mean (SD)	13.1 (13.1)	5.4 (3.6)	7.7 (6.3)	15.4 (13.6)
Contralesional paresis (MRC scale), median (range)	4 (1–5)	4 (2–5)	4 (1–5)	4 (3–4)
Tilt of SVV (absolute values in degrees), mean (SD)	0.9 (0.7)	4.4 (1.3) contra	0.8 (0.7)	5.1 (2.1) contra
Ocular torsion (% present)	0	0	0	0
No patients tested (% present)	22	–	–	–
Skew deviation (% present)	0	0	0	0
Head tilt (% present)	0	0	0	0

F, female; M, male; ccm, cubic centimeters; SD, standard deviation; SVV, subjective visual vertical; contra, contralateral tilt; MRC, Medical Research Council.

### Vestibular testing

SVV as a measure of tonic vestibular otolith perception as well as HT, skew deviation, and OT were tested as previously described (Dieterich and Brandt 1993; Brandt et al. 1994). A mean binocularly determined deviation of more than 2.5° of the static SVV was considered as abnormal (Dieterich and Brandt 1993).

### MRI scans

In all patients MRI scans were performed with a mean time interval of 5 days between lesion onset and MRI (SD 1 day). We used diffusion-weighted imaging (DWI) within the first 48 h poststroke and fluid-attenuated inversion-recovery (FLAIR) sequences when imaging was conducted 48 h or later. Lesion mapping using the Brunner–Munzel test implemented in MRicron and MRI processing using the normalization algorithm of SPM8 (http://fil.ion.ucl.ac.uk/spm/) was conducted as described previously (Rorden et al. 2007; Baier et al. 2012). To prevent a rise in the probability of familywise error, we computed a false discovery rate (FDR) correction. The results of the lesion analysis were combined with the probabilistic maps of the posterior IC with the aid of the SPM Anatomy Toolbox (Eickhoff et al. 2005; Kurth et al. 2010) whereas the anterior insular and peri-insular regions were defined by the anatomical maps provided (Bense et al. 2001; Tzourio-Mazoyer et al. 2002).

In the previously analyzed sensory data for our correlation analysis in the subgroup of 20 patients which were described above, the statistical voxelwise lesion behavior mapping (VLBM) for a binary comparison (CDT [cold detection threshold]; WDT [warm detection threshold]) was performed using the Liebermeister statistics (Rorden et al. 2007). Statistical analysis was conducted using SPSS 15.0 for Windows (SPSS Inc., Chicago, IL). For correlation analysis we used the Spearman rho analysis. In addition, we conducted a bivariate linear regression analysis to indicate prediction of one variable from another.

### Thermal perception adapted for our previous analysis

In the previous study, which was submitted for publication elsewhere, we performed quantitative sensory testing (QST) according to a protocol of the German Research Network on Neuropathic Pain (DFNS) (B. Baier, P. zu Eulenburg, C. Geber, R. Rohde, R. Rolke, C. Maihöfner, F. Birklein, M. Dieterich, unpubl. ms; Rolke et al. 2006) in 20 patients for the actual subgroup analysis (Rolke et al. 2006). We found employing a statistical VLBM with Liebermeister statistics (Rorden et al. 2007) that warm and cold perception thresholds contralateral to the stroke were strongly associated to lesions in the posterior IC (B. Baier, P. zu Eulenburg, C. Geber, R. Rohde, R. Rolke, C. Maihöfner, F. Birklein, M. Dieterich, unpubl. ms.).

## Results

Out of the 13 patients with left-sided IC lesions four patients (30%) had an abnormal (deviation of more than 2.5°) contralesional SVV deviation (mean 5.1°; mean of all left-sided lesion patients: 2.1°; SD 2.4°). In the sample of the 14 patients with right-sided lesions five patients (36%) showed contralesional abnormal SVV tilt with mean 4.4° (mean of all right-sided lesion patients: 2.2°; SD 1.9°) (Table 1). In right- and left-sided lesion patients no abnormal ipsilesional SVV deviation was observed. There is no difference between the right- and left-sided patients with regard to extent and frequency of SVV tilt (extent: unpaired *t*-test *P* = 0.96; frequency: *χ*²-test *P* = 0.79). None of the patients showed other signs of otolith dysfunction such as OT, skew deviation, or HT.

In patients with right-sided lesions the stroke area specifically associated to tilt of SVV was located at *x* = 44, *y* = 1, *z* = −9 – corresponding to the border region between the third short insular gyrus (SIG) III and the long insular gyrus (LIG) IV and at *x* = 32, *y* = 6, *z* = 10 corresponding to the border region of the white matter and right putamen. In patients with left-sided lesions the region associated with higher extent of tilt of SVV was located at *x* = −43, *y* = −10, *z* = 0 (assigned to the Ig2 with a probability of 20%) and at *x* = −37, *y* = −12, *z* = 12 (assigned to the Ig2 with a probability of 20%; to OP3 with a probability of 60%) matching the LIG IV as well as to *x* = −42, *y* = 10, *z* = 0 corresponding to the second SIG II (Fig. 1C and D).

### Subgroup analysis

**Figure 2:**
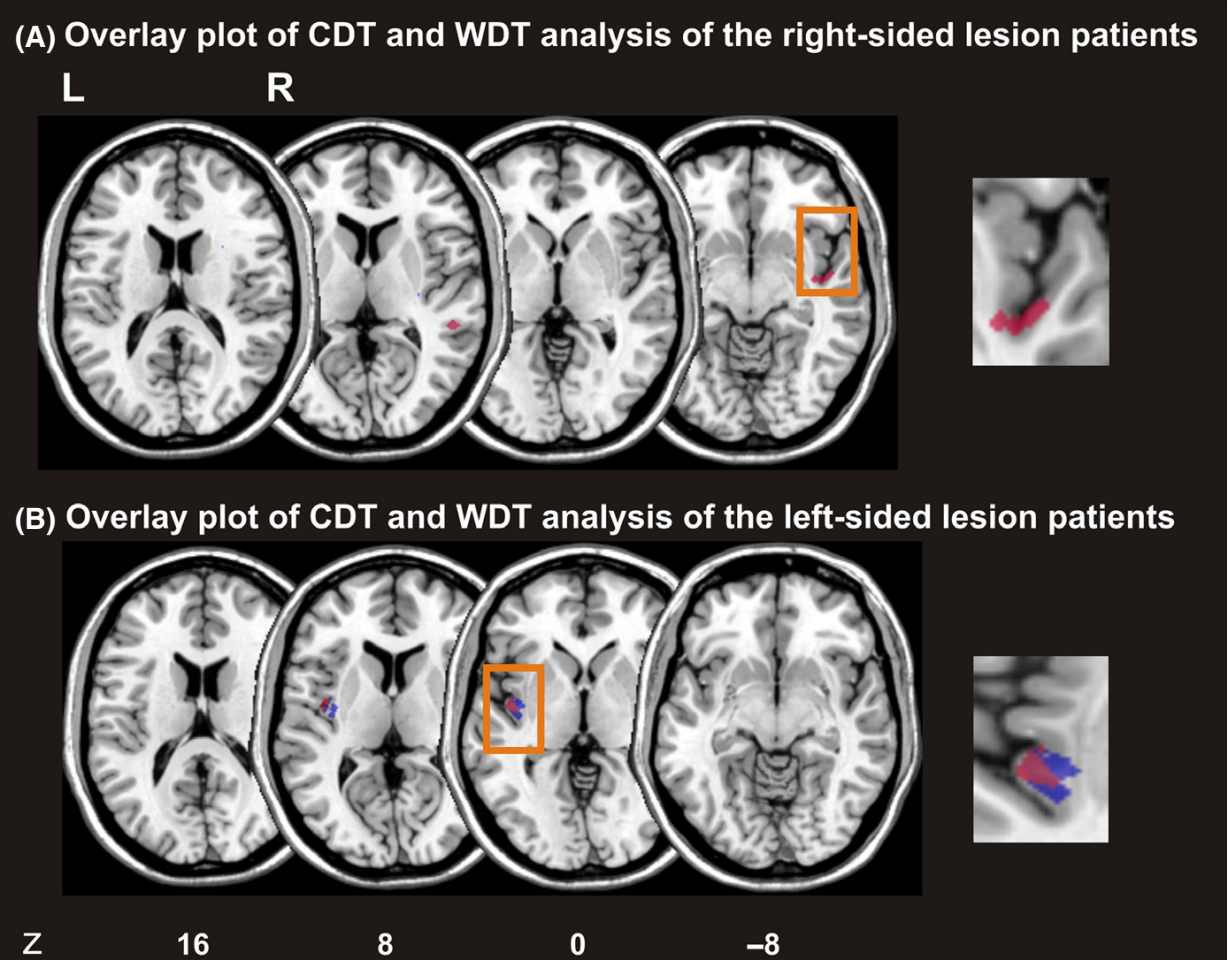
Overlay plot of the analysis of cold detection threshold (CDT) and warm detection threshold (WDT) for the right-sided lesion patients (A) and the left-sided lesion patients (B) indicating that the regions associated with pathological CDT and WDT are centered around the posterior insula. CDT corresponds to red color, WDT to blue color. For CDT and WDT (false discovery rate [FDR] corrected *P* < 0.05) Liebermeister-statistics was applied (Rorden et al. 2007).

To investigate whether there was an association between the extent of the vestibular disturbance (tilt of SVV) and the perception thermal stimuli, we reanalyzed the available data of 20 patients from our previous study (B. Baier, P. zu Eulenburg, C. Geber, R. Rohde, R. Rolke, C. Maihöfner, F. Birklein, M. Dieterich, unpubl. ms.). In these patients one with left-sided lesions (13%) had an abnormal contralesional SVV deviation (3.8°), and four of the 12 right-sided lesion patients (33%) (mean 4.5°; SD ± 1.5°). The area mainly associated with CDT and WDT was located at the LIG (Fig. 2). We now used the temperature perception results from our previous study and performed a correlation analysis and bivariate linear regression of temperature perception with SVV. There was a positive correlation between tilt of SVV and WDTs (*r*_s_ = 0.471; *P* = 0.043) and CDTs (*r*_s_ = 0.575; *P* = 0.01), showing that patients with severe vestibular dysfunction have more significant cold and warm perception deficits on the side contralateral to the stroke. Bivariate linear regression verified this correlation showing significant data for CDTs (*F* (1,17) = 8.397, *P* < 0.01) and WDTs (*F* (1,17) = 4.838, *P* < 0.05) (adjusted *R*² for CDT: 0.291; for WDT: 0.176).

## Discussion

The present data show that patients with strokes of the IC and peri-insular regions could have contralesional deviations in the perception of verticality but do not show any further pathological signs of otolith dysfunction. Thus, lesions of the IC alone do not seem to play exclusively a role in abnormal tilt of SVV, at least the peri-insular surroundings might also have to be affected (Baier et al. 2012; zu Eulenburg et al. 2012). The incidence of SVV tilts was lower than the previously reported half of the patients with SVV tilts and infarctions in the MCA territory (Brandt et al. 1994; Yelnik et al. 2002). Brandt et al. (1994) data, however, are not necessarily contradictory as these authors investigated larger strokes while we focused on small strokes affecting the IC. Obviously, lesions outside the IC, for example, the superior temporal gyrus or the inferior frontal gyrus might also be important for the perception of verticality (Brandt et al. 1994; Baier et al. 2012). Thus, a possible explanation could be that larger lesions affecting more parts of the vestibular network might lead to a more severe tilt of SVV in a higher percentage of patients. The brain lesions associated with tilt of SVV in the current study are centered at the IC and adjacent OP, probably due to projection fibers. This finding confirms previous data indicating that not only the IC but also its surrounding regions play a role in SVV tilts (Brandt et al. 1994; Baier et al. 2012; zu Eulenburg et al. 2012; Lopez et al. 2012). As a conclusion the posterior part of the IC but also surrounding regions are important brain regions for conversion of otolith signals to behavior. Interestingly, the fact that no difference was found with regard to frequency and extent of tilt of SVV in right- and left-sided lesion patients seems astonishing considering the fact that on one hand previous data report about a dominance of the right-hemisphere in spatial perception (Pérennou et al. 2008). On the other hand older data found tilt of SVV in right- and left-sided lesions patients as well (Brandt et al. 1994). Thus, considering present and previous data assessing tilt of SVV showing no difference of tilt between patients with right- and left-sided lesion patients it seems that perception of verticality is not a lateralized phenomenon.

The observation that all the other signs of otolith dysfunction did not show any pathology can be explained by the lesion location in relation to the site of the vestibular pathways. The vestibulo-ocular reflex (VOR) mediates vestibular signals from the vestibular end organ via the vestibular nucleus to the ocular motor nuclei and integration centers in the pontomesencephalic brainstem (interstitial nucleus of Cajal, INC). This reflex is responsible for the rapid coordination of the two eyes during head and body movements. A deficient VOR causes a misalignment of the eyes, that is, skew deviation and OT. The most rostral structures for eliciting this misalignment are therefore located in the rostral midbrain (INC) (Brandt et al. 1994). Therefore, the triad of ocular tilt reaction (OT, skew deviation, HT) is elicited at the infratentorial level as a brainstem sign. Lesions in the ascending pathways from the brainstem to the vestibular cortex in the insula affect only the perception of verticality – as in our study.

**Figure 3:**
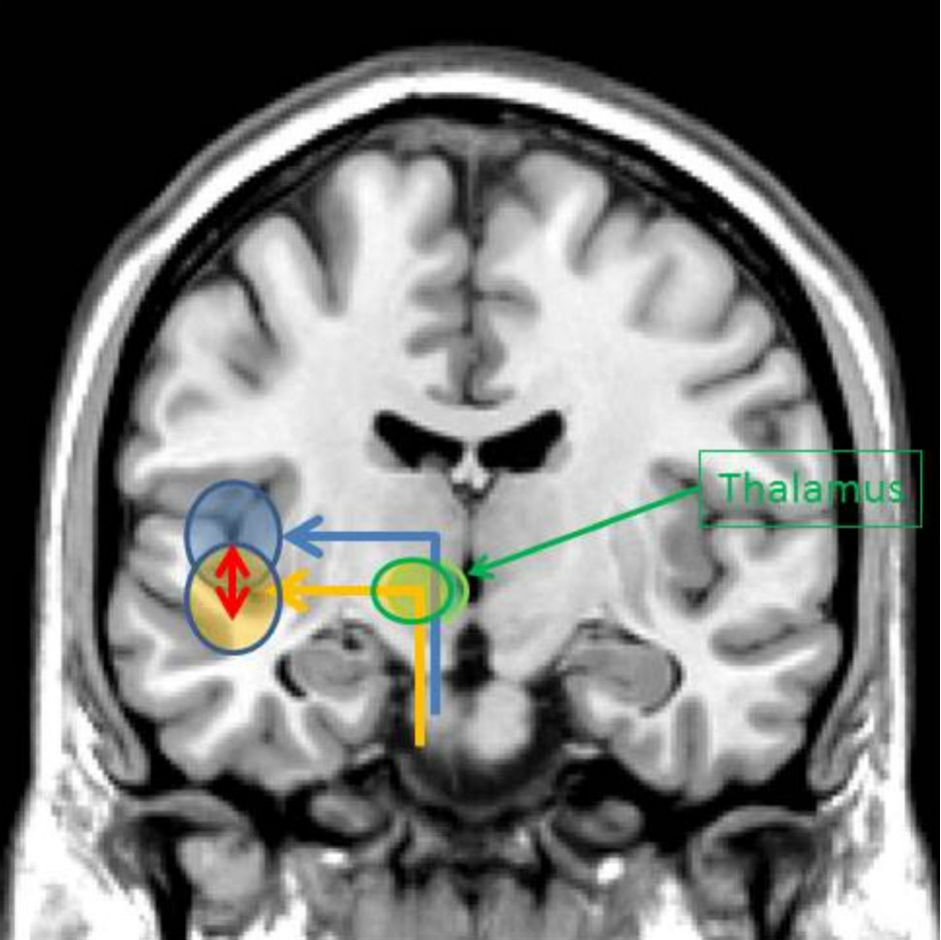
Simplified schematic drawing of central structures involved in the processing of vestibular and thermal information reaching the insular cortex as multisensory region via the thalamus. Intrainsular connections between vestibular (blue) and somatosensory signals (yellow) might lead to homeostasis and might be the basis for vestibular–somatosensory interaction (red arrow).

Our reanalysis of a possible conjunction of the tilt of SVV and thermal perception indicates that the severity of vestibular and temperature sensory deficits in acute IC stroke might be associated. The simultaneous impairment of temperature perception and tilt of SVV suggests that multisensory input converge in the IC. Our findings support the notion that the IC – and in particular its posterior part – is a primary sensory brain region integrating the different sensations. Previous data also support a close interaction between somatosensory signals such as pain and vestibular stimulation (McGeoch et al. 2008, 2009; Ferrè et al. 2012). This might be caused by the activation of posterior IC neurons leading to interdependent suppression of either feeling – finally to maintain homeostasis (Fig. 3). These manifold interactions in the IC might be the basis for the multisensory deficits often observed after IC stroke.
